# Estimating Cardiovascular Risk in HIV-Infected Patients

**DOI:** 10.36660/abc.20190747

**Published:** 2020-01

**Authors:** Marcio Sommer Bittencourt

**Affiliations:** 1 Centro de Pesquisa Clínica e Epidemiológica - Hospital Universitário da Universidade de São Paulo, São Paulo, SP - Brazil; 2 Hospital Israelita Albert Einstein, São Paulo, SP - Brazil; 3 DASA, São Paulo, SP - Brazil

**Keywords:** Carotid Artery Diseases, Acquired Immunodeficiency Syndrome/complications, HIV, Antiretroviral Therapy, Highly Active, Risk Factors

From the initial reports of HIV infections in 1981, substantial progress in the prevention, detection and treatment have led to a marked reduction in mortality. Among them, the use of highly active antiretroviral therapy (HAART) with the combination of multiple antiretroviral drugs acting in different phases of the viral cycle resulted in impressive reductions in transmission and infectious complications. This increase in survival led to a large and growing population of persons living with HIV (PLWH) as a chronic condition in a setting where other non-infectious complications became more common.

Among those novel conditions affecting PLWH, cardiovascular complications became the leading cause of morbidity and mortality, at least in countries with adequate access to care and widespread use of HAART.^1^ The increase in cardiovascular risk in PLWH is multifactorial. First, studies suggest that PLWH are more likely to be diabetics and smokers.^2^ Second, those individuals are more likely to have higher triglycerides and lower HDL-C, though LDL-C and total cholesterol were also reduced. It also seems possible that HIV infection is responsible for increases in blood pressure and glucose levels by different mechanisms.^3^ However, considerable evidence supports the concept that traditional risk factors do not fully account for the higher cardiovascular risk in those individuals,^4^ as the changes in the cardiovascular system in PWLH is multifactorial and at least partially mediated by inflammation and immune activation, leading to endothelial dysfunction, changes in vascular elasticity, coagulation.^5^

Moreover, HIV treatment with HAART may also be responsible for a considerable increase in cardiovascular risk in this population. Previous studies suggest that longer HAART duration is associated with hypertension and lipid abnormalities, particularly for first-generation protease inhibitors and non-nucleoside reverse transcriptase inhibitors.

With such abundant evidence that the spectrum of cardiovascular risk in PLWH is not similar to the usual primary prevention patient, it should come as no surprise that the traditional risk calculators such as the Framingham risk score,^6^ do not perform as expected in this population. In order to address this issue, investigators from the data-collection on adverse effects of anti-HIV drugs (D:A:D) study derived additional risk equations including HIV-related aspects such as CD4 count and type of HAART drug use to improve risk prediction in this population.^4^ Since retrieval of information related to prior HAART therapies might be challenging in real practice, an updated reduced version of the D:A:D score restricting the input related to HIV to CD4 levels has but recently published.

In the present issue of the ABC Cardiol, Silva et al.,^7^ further, evaluate the performance of this novel reduced D:A:D score in a Brazilian population comparing it to the traditional Framingham risk score in 71 PLWH who underwent carotid intima-media thickness (cIMT) for the evaluation of subclinical atherosclerosis.^7^ The authors demonstrate that both scores correlate well with the cIMT. However, individuals with low and intermediated risk in the Framingham risk score were more likely to have documented subclinical atherosclerosis in cIMT (20% vs. 6.7% in low risk and 62.5% vs. 30.8% for intermediate risk), indicating that the use of the Framingham risk scores results in underestimation of the prevalence of subclinical disease in low and intermediate risk individuals. Those findings have important implications for practice as a substantial proportion of true high-risk individuals among PLWH would remain untreated or undertreated if the cardiovascular risk is estimated only used traditional scores such as Framingham. Although the current data cannot be interpreted as a complete validation of the D:A:D score in a Brazilian population, it certainly provides evidence to support that the concept that PLWH are of higher risk for atherosclerosis and the estimation of risk in this population is more adequately performed using the D:A:D score than other scores developed for the general population.
